# Assessing the educational impact and quality of medical microvideos on TikTok: the case of Latin America

**DOI:** 10.1080/10872981.2025.2474129

**Published:** 2025-03-08

**Authors:** Juan S. Izquierdo-Condoy, Marlon Arias-Intriago, Melizza Mosquera-Quiñónez, Fernando P. Melgar Muñoz, Mariana Jiménez-Ascanio, Valentina Loaiza-Guevara, Esteban Ortiz-Prado

**Affiliations:** a One Health Research Group, Faculty of Medicine, Universidad de las Américas, Quito, Ecuador; b Facultad de Ciencias de la Salud, Universidad, Pontificia Universidad Javeriana, Bogotá, Colombia; c Facultad de Medicina, Fundación Universitaria Autónoma de las Américas, Pereira, Colombia

**Keywords:** Medical education, TikTok, Microlearning, health profession, Latin America

## Abstract

**Background:**

Social media use in medical education has surged, with YouTube and Facebook leading before COVID-19. Recently, TikTok has drawn young learners, expanding access but often lacking alignment with formal curricula and quality standards.

**Objectives:**

This study aims to analyze the quality of academic medical content on TikTok within the Latin American context, focusing on the most-viewed Spanish-language accounts.

**Materials and methods:**

This cross-sectional study analyzed medical education microvideos on TikTok through a systematic search conducted on 1 March 2024, using the keywords ‘medical education’ and ‘medical review.’ The search yielded 300 microvideos, from which the 100 most-viewed were selected. The 13 most popular accounts were identified, and their top three most-viewed microvideos were analyzed, resulting in a final sample of 39 microvideos. Popularity was measured through views, likes, and the Viewability Index (VPI), while educational quality was assessed using the JAMA Benchmark Criteria, which evaluates authorship, attribution, disclosure, and validity.

**Results:**

The majority of accounts (69.2%) were male-created, with 30.8% based in Mexico and Peru. Physicians comprised 53.8% of content creators, followed by medical students (23.1%). General medical education was the main focus (69.2%), with general medicine as the most common topic (76.9%). The dataset included 39 microvideos, averaging 1,653,677 views, and #medicina was the most frequently used hashtag. Popularity metrics strongly correlated with engagement metrics (comments, shares) but weakly with favorites. Educational quality scores were low, with only two accounts scoring 1 out of 4 points on the JAMA Benchmark.

**Conclusion:**

TikTok’s engagement metrics amplify popular medical microvideos among Spanish-speaking users but do not reliably reflect educational quality, raising concerns about misinformation. ‘Favorites’ may serve as a more accurate indicator of perceived informational value. Standardized assessment tools should incorporate both engagement and quality metrics to improve content reliability and accessibility to evidence-based medical information.

## Introduction

Medical education has undergone significant transformation, shifting from cadaver-based practice and intensive textbook study to interactive digital platforms that enhance accessibility and engagement [1]. Early digital resources, such as the Merck Manuals online and digitized medical journals, were instrumental in this evolution, expanding the reach of medical knowledge [2]. Social media platforms, notably YouTube, soon followed, offering access to expert medical content on a global scale [3,4].

During the COVID-19 pandemic, social media use in medical education surged, with Instagram, Twitter, and especially TikTok providing real-time knowledge sharing and appealing to younger audiences with short, dynamic video content [5]. TikTok, with approximately 1.8 million active monthly users – many in the 18–24 age group – has emerged as a particularly influential platform in this educational landscape [6,7]. Notably, medical students are not only consuming TikTok content but are actively generating it, contributing to the platform’s growing role in medical education [6,7]. During the pandemic, TikTok highlighted its educational potential through initiatives like #LearnOnTikTok, which promoted knowledge exchange and provided resources for students, professionals, and educators alike [8,9].

Despite TikTok’s popularity and its potential to make medical education accessible across geographical boundaries, foster interactive learning, and support asynchronous study, it also has notable limitations [10]. Concerns include the challenges of balancing engagement with educational depth and the risk of oversimplifying complex medical concepts [8,11,12].

Although studies in other regions underscore TikTok’s potential in medical education [8,13], there remains limited research focused on Latin America, where the platform’s role is underexplored and largely absent from formal medical training programs. To address this gap, this study uses a cross-sectional descriptive research approach to analyze TikTok’s role in Latin American medical education, by examining medical-related content on the most-viewed Spanish-language accounts.

## Hypothesis

We hypothesize that while TikTok offers significant engagement potential, the educational quality of its content may be limited, underscoring the need for quality standards and further integration into formal medical training.

## Materials and methods

### Study design

This study employed a cross-sectional descriptive research approach, conducted via internet searches on the social network TikTok.

### Research design and population

The study was conducted on the TikTok platform, focusing on microvideos related to medical education. A systematic search was performed one week before 1 March 2024 (from 23 February 2024, to 1 March 2024). To minimize search bias, browser cookies were deleted on all devices prior to the search. Using the keywords ‘medical education’ and ‘medical review,’ the search yielded 300 microvideos. From this initial pool, the 100 most-viewed microvideos were selected for detailed analysis. These videos were posted between October 2021 and January 2024, covering a 27-month span period. Additionally, the most popular TikTok accounts responsible for these microvideos were identified for further evaluation.

The study population included TikTok accounts and microvideos related to medical education, created by medical professionals, medical students, and other health-related content creators. The primary exposure of interest was the medical education content on TikTok, including both the TikTok accounts and the microvideos they posted. The primary outcomes measured were the popularity and educational quality of the microvideos. Popularity was assessed based on the number of views, likes, and the Viewability Index (VPI), while educational quality was evaluated using the JAMA Benchmark Criteria, a set of standards developed by the Journal of the American Medical Association (JAMA) to assess the credibility and reliability of health-related information (JAMA, 1998) [14].

To ensure the selection of relevant content, the following criteria were applied. Inclusion Criteria: Only microvideos that ranked among the top 100 most-viewed, were in Spanish, contained audio, and were directly related to professional medical education – specifically content designed to meet the learning needs of medical students and physicians – were included. Exclusion Criteria: Videos failing to meet these criteria, such as those not produced in Spanish, which led to the exclusion of medical content from Brazilian creators in Portuguese, as well as videos lacking audio, unrelated to professional medical education, or classified as advertisements, were excluded from the analysis. After applying these selection criteria, the top 13 most popular accounts were identified for final evaluation.

### Data source

Data was collected from the TikTok platform using internet searches and the platform’s built-in metrics for popularity, including the number of views, likes, and shares.

### Micro-video selection

**Figure 1.**
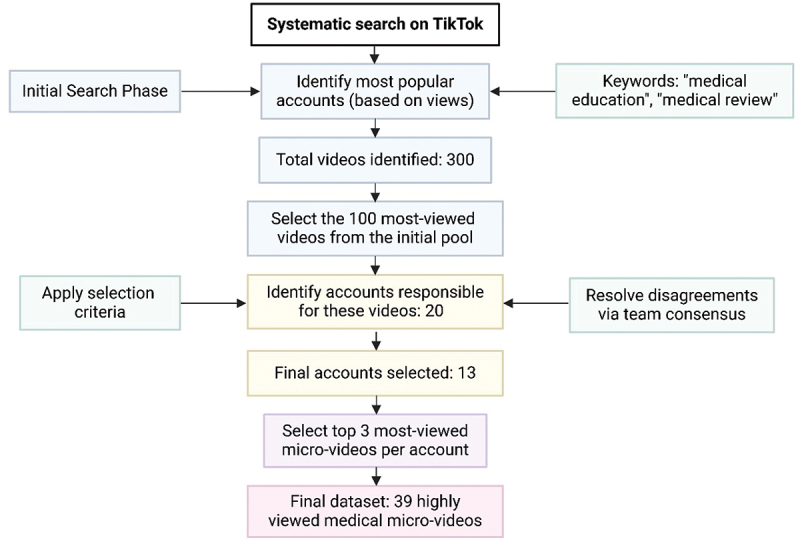
Flowchart of the medical education microvideo selection process.

A systematic search on TikTok identified 300 medical education microvideos based on view count. The 100 most-viewed were filtered to 20 accounts, then refined to 13. The top three videos from each account were selected, totaling 39 microvideos (Figure 1).

### Parameters and micro-videos evaluation

The accounts creating the microvideos were evaluated according to the following parameters: country of origin, number of followers, type of account (academics, physicians, medical students, non-medical health professionals, non-health professionals, commerce), academic qualification of the content creator (general practitioner, specialist physician, subspecialist physician, physician with a master’s degree, physician with a doctorate, unspecified), and the subject of the account (general medicine or educational content specialized in the medical area). The microvideos were evaluated using metrics such as duration in seconds, number of views, number of likes, and the Viewability Index (VPI), calculated as the ratio of views to likes. The microvideos were also evaluated based on ‘value’ metrics, including the number of comments (indicator of intercommunication), number of bookmarks (indicator of content saved for later review), and number of shares (indicator of distribution among users).

To assess the reliability, accuracy, and educational value of the information presented in the selected videos, the JAMA Benchmark criteria were applied. The JAMA Benchmark criteria use a 4-point scale evaluating authorship, attribution, disclosure, and validity. Each microvideo was evaluated and scored on this scale, with scores ranging from 0 to 4 points [7,15,16]. Videos achieving a score of 3 or more were classified as ‘reliable.’

### Statistical analysis

Descriptive statistics were presented as frequency (n) and percentages (%) for categorical variables. For numerical variables, the distribution was evaluated using the Shapiro-Wilk test, and the mean and standard deviation were used for their description. Pearson’s correlation test was used to evaluate associations between quantitative variables (popularity metrics versus content value metrics). A p-value of < .05 was considered statistically significant. Statistical analyses were performed with SPSS Statistics 16.0 (SPSS Inc., Chicago, IL).

## Results

### Account analysis

A total of 13 accounts were fully analyzed. The results show that most of the accounts (69.2%, *n* = 9) were created by male content creators. In terms of residence, 30.8% (*n* = 4) of the content creators were residents of Mexico and Peru. The predominant account type was physicians (53.8%, *n* = 7), followed by medical students (23.1%, *n* = 3). The most common academic level was general practitioner (30.8%, *n* = 4).

The focus of the accounts was predominantly on general medical education (69.2%, *n* = 9), with only 3 accounts focused on specific exam preparation. Additionally, the predominant topic was general medicine (76.9%, *n* = 10), with only 3 accounts addressing obstetrics, dentistry, and pediatrics. The number of followers ranged from 6,600,000 for account 1 to 9,501 for account 5, with a mean of 614,453.9 followers (Table 1).

**Table 1.** Demographic and professional characteristics of the accounts and their content creators.

Account Id	Number of followers	Sex	Country of residence	Type of account source	Academic level	Educational Approach of the content	Account topic
Account 1	6,600,000	Male	Colombia	Medical Doctor	Internal medicine	General	General medicine
Account 2	393,700	Female	Spain	Medical Student	NA	MIR	General medicine
Account 3	248,300	Male	Peru	Commercial	Non specified	ENAM	General medicine
Account 4	10,700	Male	Peru	Medical Doctor	Subspecialty	General	General medicine
Account 5	9,501	Female	Ecuador	Health professionals/no MD	NA	General	Obstetrics
Account 6	57,200	Male	Bolivia	Medical Doctor	General	General	General medicine
Account 7	39,200	Male	Peru	Medical Doctor	General	ENAM	General medicine
Account 8	24,500	Female	Mexico	Medical Student	NA	General	General medicine
Account 9	111,600	Male	Mexico	Medical Doctor	General	General	General medicine
Account 10	63,300	Male	Peru	Medical Student	NA	General	General medicine
Account 11	40,000	Male	Mexico	Medical Doctor	General	ENARM	General medicine
Account 12	332,700	Female	Mexico	Health professionals/no MD	NA	General	Odontology
Account 13	57,200	Male	Venezuela	Medical Doctor	Subspecialty	General	Pediatrics

MIR: resident medical intern exam; ENARM: national examination for medical residency applicants.

**Figure 2.**
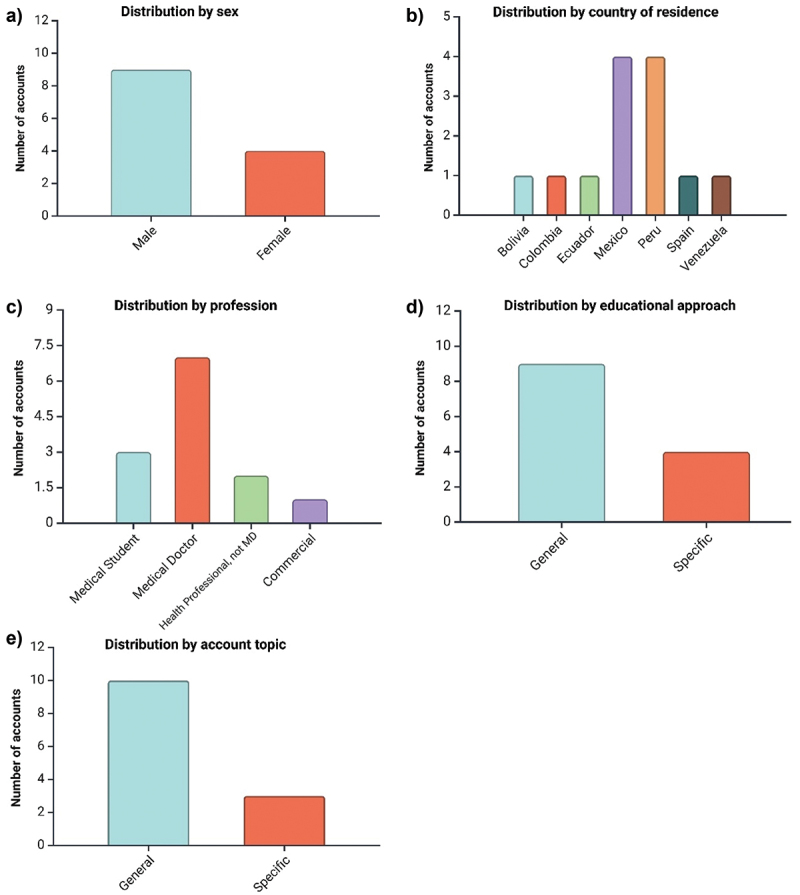
Distribution of the TikTok accounts analyzed, according to the characteristics of the content creators.

The analysis of the 13 TikTok accounts revealed that 69.2% (*n* = 9) were created by male content creators, with the remaining 30.8% (*n* = 4) by females. Geographically, the majority of content creators were from Mexico and Peru, each accounting for 30.8% (*n* = 4) of the total accounts, with the rest from Bolivia, Colombia, Ecuador, Spain, and Venezuela (Figure 2).

Physicians made up 53.8% (*n* = 7) of the creators, followed by medical students at 23.1% (*n* = 3), health professionals without an MD at 15.4% (*n* = 2), and one commercial account. Most accounts (69.2%, *n* = 9) focused on general medical education, while 30.8% (*n* = 4) targeted specific exam preparation. General medicine was the primary topic for 76.9% (*n* = 10) of the accounts, with only three accounts addressing obstetrics, dentistry, and pediatrics.

### Micro-videos hashtag analyses

In the context of medical education, a total of 134 different hashtags were identified, used 254 times across the 39 analyzed micro-videos. Among these, 96 hashtags were mentioned only once. The most frequently used hashtag was #medicina (#medicine), appearing 18 times (7.1%). This was followed by #doctor (#doctor) and #parati (#foryou), each used 9 times (3.5%).

Other notable hashtags included #ENARM, used 7 times (2.8%), and #farmacologia (#pharmacology), used 5 times (2.0%). Additionally, hashtags like #medstudent (#medstudent), #aprendecontiktok (#learnwithtiktok), and #estudiantes (#students) were used 5 times (2.0%), 4 times (1.6%), and 4 times (1.6%) respectively, highlighting their relevance as key terms in medical education content on TikTok.

**Figure 3.**
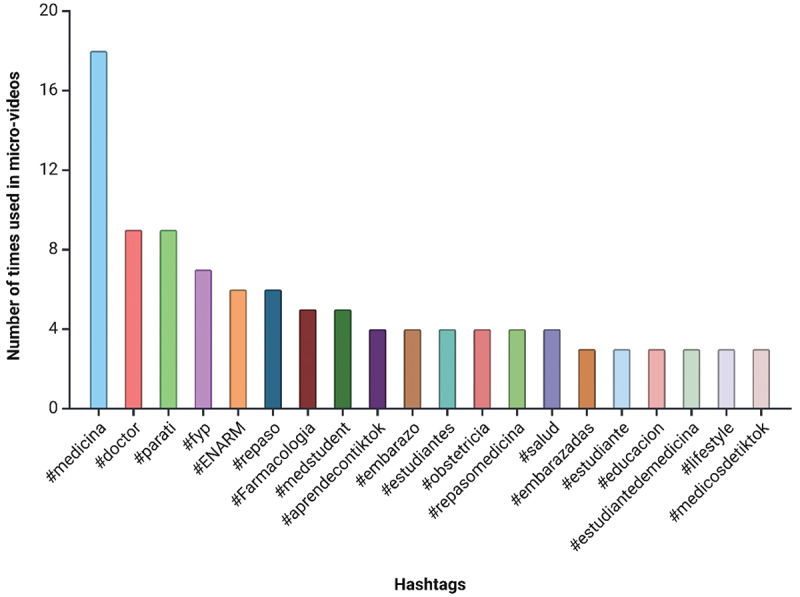
Distribution of the top 20 hashtags identified in the 39 medical education micro-videos.

The distribution of these hashtags reflects a diverse range of topics within the medical education field, with a significant focus on general medical education, exam preparation, and specific areas such as pharmacology and student engagement (Figure 3).

### Micro-videos metric analyses

The impact of the microvideos was consistent among the evaluated accounts, with Accounts 1 and 3, both male content creators from Colombia and Peru, respectively, showing the highest average number of views, exceeding one million (14,333,333 views for Account 1 and 1,833,000 views for Account 3). These accounts also showed the highest impact indicators. Account 1 had 1,016,333 likes and Account 3 had 137,833.3 likes. In terms of user interaction, Accounts 1 and 3 had the highest number of comments (11,157 and 1,061.3, respectively), significantly higher than the average number of comments for the other accounts. Account 3 had the highest average number of bookmarks as favorites (111,633.3), followed by Account 1 (85,933.3) and Account 9 (51,733.3). Similarly, these accounts had the highest average number of microvideos shared. Overall, Account 1 consistently outperformed other accounts across all metrics, demonstrating high engagement and popularity (Table 2).

**Table 2.** Micro-video metrics of the TikTok accounts analyzed.

Account Id	MV length in seconds (mean ± SD)	MV views (mean ± SD)	Number of MV likes (mean ± SD)	VPI (mean ± SD)	Number of MV comments (mean ± SD)	Number of MV marks as favorites (mean ± SD)	Number of MV shares (mean ± SD)
Account 1	135.0 (±174.1)	14,333,333.0 (±6,106,826.7)	1,016,333.0 (±236,855.1)	136,364,666,667 (± 30800,255,605)	11,157.0 (±3202.2)	85,933.3 (±54,303.1)	36,200.0 (±17,754.2)
Account 2	127.3 (±61.2)	625,000.0 (±287,982.3)	101,333.3 (±19,824.5)	667,670,933.3 (±44,003,9175.3)	251.0 (±83.5)	40,170.7 (±31,594.2)	4,286.0 (±4484.9)
Account 3	157.3 (±12.6)	1,833,000.0 (±1,665,733.2)	137,833.3 (±107,060.3)	3,715,132,333 (±5,055,547,828.4)	1,061.3 (±1,197.7)	111,633.3 (±135,195.5)	7,934.0 (±8,320.4)
Account 4	115.3 (±55.8)	222,473.7 (±228,338.6)	18,315.3 (±15,735.4)	60,695,726.22 (±63,309,960.2)	8.7 (±12.5)	4,355.3 (±3,766.2)	1,601.0 (±2,110.0)
Account 5	41.7 (±16.1)	823,366.7 (±231,717.3)	8,708.7 (±6,654.0)	71,358,353.33 (±63,775,573.2)	196.7 (±86.6)	3,507.3 (±3,454.2)	694.7 (±490.4)
Account 6	141.3 (±37.8)	1,552,266.7 (±1,340,886.9)	20,000.0 (±3,143.2)	315,089,600 (±265,682,659.9)	165.7 (±148.9)	3,914 (±2,346.4)	3,677.7 (±2,393.5)
Account 7	215.7 (±112.4)	193,233.3 (±19,918.9)	20822.7 (±22,458.1)	70,037,802.67 (±106,939,609.2)	50.0 (±40.6)	11,694 (±12,748.1)	1,313.0 (±951.5)
Account 8	73.7 (±42.3)	77,566.7 (±50,400.0)	6260.0 (±4,458.7)	6,337,857.333 (±6,439,642.7)	15.3 (±5.0)	3,119.7 (±2319.4)	454.0 (±445.5)
Account 9	63.0 (±5.6)	863,900.0 (±67,756.4)	113033.3 (±39,566.4)	983,930,333.3 (±388,617,269.2)	172.0 (±30.3)	51,733.3 (±1,698.,8)	3,901.7 (±1,072.0)
Account 10	157.7 (±16.5)	38,966.7 (±18,951.0)	2772.3 (±1,790.0)	1,302,728 (±1,281,117.5)	24.7 (±11.9)	1,185.0 (±840.6)	251.7 (±96.9)
Account 11	60.0 (±1.0)	326,433.3 (±219,945.5)	32899.7 (±23,948.6)	135,545,289.3 (±113,641,550.1)	87.0 (±54.8)	9,466.7 (±10,375.9)	1,246.0 (±1,489.7)
Account 12	68.3 (±38.9)	564,866.7 (±386,071.3)	86766.7 (±6,3942.0)	654,658,200 (±800,872,008.3)	109.7 (±89.1)	33,666.7 (±26,581.6)	3,366.7 (±3,500.2)
Account 13	118.7 (±56.9)	43,400.0 (±39,343.4)	1,756.0 (±741.1)	753,005 (±624,794.0)	385.3 (±624.1)	461.3 (±248.7)	66.0 (±48.8)
Total (mean)	113.5 (±67.9)	1,653,677.4 (± 4,034,725.4)	120,525.7 (± 273,290.8)	11,003,629,140.6 (± 37367,938,575)	1052.6 (± 3,037.1)	27,757.0 (± 49649.2)	4,999.4 (± 10518.1)

MV: microvideos; VPI: view power index; SD: standard deviation.

Considering the limited duration of TikTok microvideos, the analysis showed that the average duration per account was less than 1 minute, ranging from 41.7 seconds for account 5 to a maximum of 215.7 seconds for account 7, with an overall average of 113.5 seconds (Table 2).

The effect of popularity metrics (followers, duration, likes, views, and VPI) on the value metrics for the microvideos (comments, marks as favorites, and shares) showed that duration was not correlated with any metric (*p* > 0.05). However, the number of followers, likes, views, and VPI had strong positive correlations (r^2^ >0.8; *p* < 0.001) with the number of comments and shares. In contrast, the number of marks as favorites exhibited only weak correlations (r^2^ between 0.208 and 0.438; *p* < 0.05) (Table 3).

**Table 3.** Correlation between popularity and value metrics of TikTok’s medical education micro-videos.

	MV comments		MV marks as favorites		MV shares	
	r2	p-Value	r2	p-Value	r2	p-Value
Followers	0,962	<0,001	0,369	0,021	0,875	<0,001
MV length (seconds)	0,017	0,919	0,208	0,204	0,301	0,063
MV likes	0,961	<0,001	0,386	0,015	0,808	<0,001
MV views	0,883	<0,001	0,438	0,005	0,969	<0,001
MV VPI	0,964	<0,001	0,362	0,023	0,912	<0,001

### Micro-video quality assessment

When evaluated using the JAMA Benchmark, neither Account 1 nor Account 3 scored highly, both having a mean JAMA Benchmark score of 0 (Table 4). Only two accounts, Account 9 and Account 11, both male content creators from Mexico and medical doctors focusing on general medicine, had a mean score of 1 out of 4 points on the JAMA Benchmark scale. Among these, only Account 9 had a considerable mean number of likes (113,033.3) and bookmarks as favorites (51,733.3) (Tables 2 and 4). Additionally, from the parameters evaluated by the JAMA Benchmark scale, the only parameter in which 4 of the 13 accounts scored was ‘authorship,’ where the content creators disclosed the authorship of the sources they used for the micro-videos

**Table 4.** Quality assessment of TikTok microvideos according to JAMA Benchmark criteria.

Account Id	Authorship^a^ (mean/1)	Attribution^b^ (mean/1)	Disclosure^c^ (mean/1)	Currency^d^ (mean/1)	Total JAMA Benchmak (mean/4)
Account 1	0	0	0	0	0
Account 2	0	0	0	0	0
Account 3	0	0	0	0	0
Account 4	0,33	0	0	0	0,33
Account 5	0	0	0	0	0
Account 6	0	0	0	0	0
Account 7	0	0	0	0	0
Account 8	0,33	0	0	0	0,33
Account 9	1	0	0	0	1
Account 10	0	0	0	0	0
Account 11	1	0	0	0	1
Account 12	0	0	0	0	0
Account 13	0	0	0	0	0

^a^Authors and contributors, their affiliations, and relevant credentials should be provided; ^b^References and sources for all content should be listed clearly, and all relevant copyright information should be noted; ^c^Account “ownership” should be prominently and fully disclosed, as should any sponsorship, advertising, underwriting, commercial funding arrangements or support, or potential conflicts of interest; ^d^Dates when content was posted and updated should be indicated.

## Discussion

This study highlights TikTok’s increasing role in medical education across Latin America, where Spanish-language content from general practitioners and medical students has gained substantial reach. Among the 13 analyzed accounts, nine were created by male content creators, a notable contrast to the female predominance in Latin American medical schools [17–20]. This discrepancy may reflect broader gender dynamics in content creation and social media engagement within the region. Mexican and Peruvian creators represented the largest proportion of content producers, which aligns with their countries’ significant population sizes and active digital presence. Additionally, general practitioners were the most common type of content creators, potentially reflecting the challenges of accessing medical specialty training in the region [21]. This trend suggests that general practitioners may be leveraging TikTok to share knowledge and engage with a wider audience despite barriers to specialization.

General medical education was the predominant focus of the analyzed accounts. However, three accounts specifically targeted preparation for high-stakes medical exams, such as the MIR (Medical Intern Residency Exam) in Spain and the ENARM (National Examination for Medical Residency Applicants) in Mexico. These exams are widely recognized and highly competitive, drawing thousands of Latin American physicians annually. In 2024 alone, approximately 3,700 Latin American candidates took the MIR, while 30,000 applicants attempted the ENARM [22,23]. Given the complexity of these exams, many Latin American physicians turn to TikTok for continuous preparation and supplementary learning resources.

Regarding content creators, we observed a range from licensed physicians to non-health professionals. A study by Hasan et al. on orthopedic medical content on TikTok found that only 12.1% of all creators were doctors, while 81.8% were non-medical health providers [24]. In contrast, our study showed that 53.8% of the most popular medical education content creators were doctors, suggesting that a significant proportion of medical content is still being produced by individuals without formal medical training.

Despite the proven utility of hashtags for organizing and discovering reliable medical educational content on other social networks [25–27], their use was limited in the TikTok micro-videos analyzed in this study. Although hashtags like #MedEd and #LearnOnTikTok are commonly associated with educational content, they were absent in most of the analyzed videos. This finding underscores the need for a standardized approach to hashtag use within the medical education community to enhance content discoverability [7,28,29].

Audience engagement metrics serve as key indicators of content reach and effectiveness in short-form video platforms. In this study, the 39 analyzed micro-videos from the 13 most popular medical accounts averaged over 1.6 million views and 120,000 likes, highlighting TikTok’s capacity for widespread dissemination of medical content. TikTok distinguishes itself from other social media platforms through its user-driven algorithm, which rapidly curates and distributes content based on individual user interactions. This algorithm favors engagement-driven ‘viral’ and ‘trending’ content, enabling information to be uploaded and shared within seconds [16,30]. Beyond view and like counts, TikTok provides additional engagement features, such as the ability to save videos as favorites or share them with others. These features offer deeper insights into content impact. Users may like a video due to its visual appeal, editing quality, or topic, while they are more likely to save or share videos that they perceive as educationally valuable. Notably, our findings indicate that while likes, views, and VPI serve as strong indicators of popularity, they do not necessarily correlate with the number of saves as favorites.

Interestingly, some less popular videos generated a higher number of favorites, suggesting that saving as favorites operates independently of likes, shares, and comments. This highlights the potential of favorites as a key metric for evaluating the perceived value of micro-videos. Another critical engagement indicator is the number of comments, which reflects direct interaction between users and content creators [31]. In our analysis, comment frequency showed a strong positive correlation with overall engagement, a trend consistent with findings by Sun et al., who observed a similar relationship between likes and comments in TikTok videos related to gallstone disease [32].

The assessment of medical content quality on social media has led to the development of tools like the DISCERN instrument and the Global Quality Score (GQS), which primarily evaluate patient-directed content rather than materials for medical students and healthcare professionals [15,32]. Due to these limitations, this study used the JAMA Benchmark as an objective tool to evaluate TikTok’s medical education content, despite its original focus on patient information [33]. The JAMA Benchmark measures content quality based on authorship, attribution, disclosure, and currency, assigning a binary score to each criterion for a total of four points. Previous research has linked higher JAMA scores to increased user engagement. A study by Cai et al. on gestational diabetes content in China reported an average JAMA score of 2.33 ± 0.72, higher than those observed in this study. Cai et al. also found a strong positive correlation between JAMA scores and engagement metrics, including likes, comments, and shares (*p* < 0.001), supporting the association between content quality and user interaction [34].

Despite TikTok’s ability to generate high engagement, this study reveals a clear disconnect between the popularity of medical content and its educational quality. Accounts from Mexico, Colombia, and Peru obtained low scores on the JAMA Benchmark, suggesting that the platform’s algorithm prioritizes engagement over accuracy, potentially amplifying unreliable medical information. These findings align with previous research indicating that high-quality health information on platforms such as YouTube and TikTok is often overshadowed by visually appealing or sensational content [35]. A study analyzing gallstone-related TikTok content demonstrated that medically accurate information frequently received less attention than videos designed primarily for entertainment, further highlighting the challenge of ensuring reliable medical education in the digital landscape [32].

Video-based learning has demonstrated considerable potential in medical education. A meta-analysis by Morgado et al., which examined 40 studies across various regions, found that video-based learning significantly enhances knowledge acquisition in dentistry, with moderate benefits also observed in medical and nursing education for both knowledge retention and skill development [36]. However, implementing video-based learning in low- and middle-income countries presents unique challenges. Limited internet access remains a significant barrier, restricting equitable access to educational resources in underserved areas [37,38].

Microlearning, which delivers educational content in short, high-yield segments, has proven effective in improving knowledge retention and increasing learner confidence. TikTok’s micro-video format provides instant access to educational material, enabling students and professionals to reinforce their medical knowledge efficiently. However, maintaining the quality and accuracy of these resources is crucial to maximizing their educational impact [13,39]. These findings highlight the need for greater involvement from medical educators and institutions on these platforms to ensure the availability of reliable and engaging medical education content [13,40].

The negative correlation between video quality and user engagement underscores the broader challenge of ensuring the accuracy of health information in the digital age, particularly on TikTok.

The negative correlation between video quality and user engagement highlights the challenge of ensuring accurate health information on TikTok. This study underscores the need for a specialized quality assessment tool for medical education content, integrating both quality indicators and engagement metrics such as likes, views, VPI, and favorites. Since popularity does not reliably indicate educational value, favorites may serve as a more meaningful metric for assessing content relevance. A tailored assessment tool incorporating these factors would enhance the evaluation of TikTok’s role in medical education. As digital platforms shape learning strategies, maintaining content accuracy and adherence to scientific and academic standards is essential for effective integration into healthcare education.

A significant challenge in using social media for medical education stems from the role of influencers, whose content is often not standardized or rigorously evaluated. The lack of quality control and objective assessment increases the risk of misinformation, particularly when influencers prioritize engagement or monetization over accuracy [41–43]. This issue is especially relevant on TikTok, where the rapid, user-driven format often lacks stringent content oversight. While TikTok prohibits health misinformation that could pose direct harm, much of the medical content remains unregulated regarding quality, accuracy, and potential conflicts of interest. This highlights the need for accessible, evidence-based health information to support emerging healthcare professionals. Establishing an infodemiology framework to monitor and assess health information quality on social media could help mitigate these challenges and ensure that digital education tools align with best practices in medical learning [44].

## Strengths and limitations

This study provides the first in-depth analysis of Spanish-language micro-video content on TikTok aimed at Latin American audiences. It highlights the substantial reach of these videos, many of which accumulate millions of views, demonstrating their potential for engagement and influence. While most content is created by healthcare professionals and focuses on general medical education, concerns arise regarding its scientific rigor. The findings underscore the low quality of popular Spanish-language medical content on TikTok, particularly in areas such as information reliability, ownership disclosure, and transparency. These results emphasize the need for improved monitoring and verification of medical information on the platform [35]. Additionally, strategies are required to enhance the visibility and appeal of high-quality educational content to counter misinformation.

Several limitations should be considered when interpreting the findings. First, the analysis focused on the most popular Spanish-language content related to medical education on TikTok, which may not fully represent the diversity of medical education materials available on the platform. TikTok’s algorithm influences content selection based on user interactions, potentially excluding high-quality educational videos with lower engagement.

The study relied on publicly available engagement metrics, including likes, views, the VPI, and the number of favorites, to assess content popularity and perceived value. While these metrics offer insight into user interaction, they do not directly measure educational quality or content accuracy. The absence of standardized tools specifically designed to evaluate medical education content in TikTok micro-videos limited a comprehensive quality assessment. Although the JAMA Benchmark provides a validated framework for evaluating medical content, it was originally developed for patient education materials and may not fully capture the characteristics of content intended for medical students and professionals.

Cultural and regional differences in the creation and consumption of medical education content were not analyzed, limiting the generalizability of the findings beyond Spanish-speaking countries, particularly Mexico and Peru. The exclusion of Portuguese-language content, particularly from Brazil, further restricts the study’s applicability across all Latin America. Future research should incorporate Portuguese-language micro-videos to provide a more comprehensive understanding of TikTok’s role in medical education across the region.

Additionally, the cross-sectional design prevents causal inferences regarding the relationship between content popularity and educational quality. Longitudinal studies are needed to examine how engagement metrics and content quality evolve over time and their impact on medical education outcomes.

## Conclusions

Our findings suggest that while TikTok’s algorithmic features and engagement metrics amplify the reach of popular microvideos, they do not inherently measure or reflect educational quality. This misalignment raises concerns about the potential spread of low-quality medical information, particularly for healthcare professionals in training. However, as medical doctors are trained in evidence-based practice, the extent to which this poses a significant public health risk remains uncertain. Notably, ‘favorites’ emerged as an engagement metric that appears less influenced by overall popularity, potentially serving as a more accurate reflection of users’ perceived informational value.

Although standardized assessment tools for evaluating medical education content on social media could help improve content quality, their effectiveness may be constrained by TikTok’s platform design and algorithms. Future research should explore frameworks that integrate both engagement and content quality metrics to enhance the accessibility and prioritization of evidence-based medical information.
